# Supplementary material for: Discriminating between negative cooperativity and ligand binding to independent sites using pre-equilibrium properties of binding curves
Source: PLoS Comput Biol. 2020 Jun 4;16(6):e1007929. doi: 10.1371/journal.pcbi.1007929 (PMC7297384; doi:10.1371/journal.pcbi.1007929)
Supplement: S1 Text — (DOCX) [file pcbi.1007929.s001.docx]

**Supplementary Information**

Discriminating between negative cooperativity and ligand binding to independent sites using pre- equilibrium properties of binding curves

Federico Sevlever, Juan Pablo Di Bella, Alejandra C. Ventura

Index

[1. Analytical Calculations 2](#_Toc36626947)

[1.1 Non-identifiability manifold 2](#_Toc36626948)

[1.2 Features of the $\mathbf{DynR}$ versus time curve. 4](#_Toc36626949)

[1.2a. The cooperativity factor $\omega$ (in **NC**) defines the relation between $K_{01}$ and $K_{10}$ (in **IB**). 4](#_Toc36626950)

[1.2b Equilibrium **NC** dynamic range decreases with $\omega$. 4](#_Toc36626951)

[1.2c Equilibrium dynamic range comparison. 5](#_Toc36626952)

[1.2d Early **NC** dynamic range is controlled by $\omega$. 6](#_Toc36626953)

[1.2e Early **IB** dynamic range is controlled by $\boldsymbol{k}_{\mathbf{10}}$/$\boldsymbol{k}_{\mathbf{01}}$. 7](#_Toc36626954)

[1.2f **IB** dynamic range regimes are controlled by $\boldsymbol{k}_{\mathbf{10}}$/$\boldsymbol{k}_{\mathbf{01}}$. 7](#_Toc36626955)

[2. Testing the algorithm: Simulated and experimental data 10](#_Toc36626956)

[2.1 Thresholds in the algorithm. 10](#_Toc36626957)

[2.2 The algorithm applied to experimental data. 14](#_Toc36626958)

[2.3 The Two-Site Model (**TS**). 16](#_Toc36626959)

[2.3a Comparison with **IB** and **NC** 16](#_Toc36626960)

[2.3b Analogy between **TS** and **NC** 17](#_Toc36626961)

[2.3c Degrees of freedom 18](#_Toc36626962)

[2.3d Analogy between **TS** and **IB** is not possible 18](#_Toc36626963)

[2.3e Equilibrium dose-response curves 18](#_Toc36626964)

[2.3f Conclusions 19](#_Toc36626965)

[3. All the parameter values used in the paper and this supplement. 19](#_Toc36626966)

[4. References 20](#_Toc36626967)

# 1. Analytical Calculations

## 1.1 Non-identifiability manifold

The expressions in Eqs. (3) and (4) define a manifold in the parameters space in which **IB** and **NC** models give the same equilibrium dose-response curve. This manifold is plotted in Fig AA, in the $K$-$K_{10}$-$K_{01}$ space, with $\omega$ in a color scale. As $\omega$ decreases from 1 in **NC** model (moving from **p** to **q**), the corresponding **IB** set must increase the difference between $K_{10}$ and $K_{01}$to reproduce the same dose-response curve (see Section 2 in this document). For any pair $\left( K,\omega\right)$, there are two sets $\left( K_{10},K_{01} \right)$. This arises from a manifold reflection symmetry along the vertical plane defined by $K_{10}=K_{01}$, which represents both model’s symmetry in swapping binding sites ($01\boldsymbol{\leftrightarrow}10$). It can be seen also that $\omega$ defines a proportional relation between $K_{10}$ and $K_{01}$ of its indistinguishable **IB** model (see Section 2). This means that, for a point **p** in the symmetry plane where $\omega=1$, the three ratios ($K$, $K_{10}$, $K_{01}$) are equal, making both models the same in equilibrium (a receptor with 2 identical sites). On the other hand, moving to lower $\omega$, like in point **q**, $K_{10}<K_{01}$ and its difference increases with lower $\omega$, expanding the global $\mathbf{DynR}$ as shown in the main text.

We can also compare equilibrium dose response curves for the same path **p** to **q**. These are shown in Fig AB and are the same as in Fig 1C in the main text. Going from **p** to **q** means to go through all curves from **IB** or from **NC** with $\omega<1$. We can interpret the middle flat region that appears for $\boldsymbol{\theta}=\frac{1}{2}$ in both models. In **NC**, as $\omega$ decreases, the second binding is harder to occur and needs an increase in ligand concentration to happen, increasing the 𝐃𝐲𝐧𝐑. In **IB**, a decrease in $\omega$ means the non-identifiable values of ($K_{10}$, $K_{01}$) are more distant between each other to reproduce the same dose response curve. The middle flat region appears because the more affine site (10) is already saturated but the less affine site (01) needs a high increase in ligand concentration for binding to occur, increasing .

Going back from **q** to **p** makes a simple explanation on why the non-identifiability problem does not arise for positive cooperativity. This path requires, from **IB**, to bring $K_{10}$ and $K_{01}$ closer and, from **NC**, to increase $\omega$, as $\mathbf{DynR}$ from both decreases. But when we reach **p**, we have $\omega=1$, $K_{10}=K_{01}$ and both affinities cannot be more similar and $\mathbf{DynR}$ for the **IB** model cannot be reduced. If we keep increasing $K_{01}$ over $K_{10}$, the dose-response curves will stay the same that going backwards because of the symmetry in swapping binding sites and $\mathbf{DynR}$ will increase. But it is possible to increase $\omega$ leading to positive cooperativity. Here, the first binding to any of both equal sites makes the other site more affine and more likely to occur than the first one, decreasing even more the$\mathbf{DynR}$ as less ligand concentration is required. This phenomenon is impossible to reproduce just choosing two different affinities in **IB** (see Section S3).

**
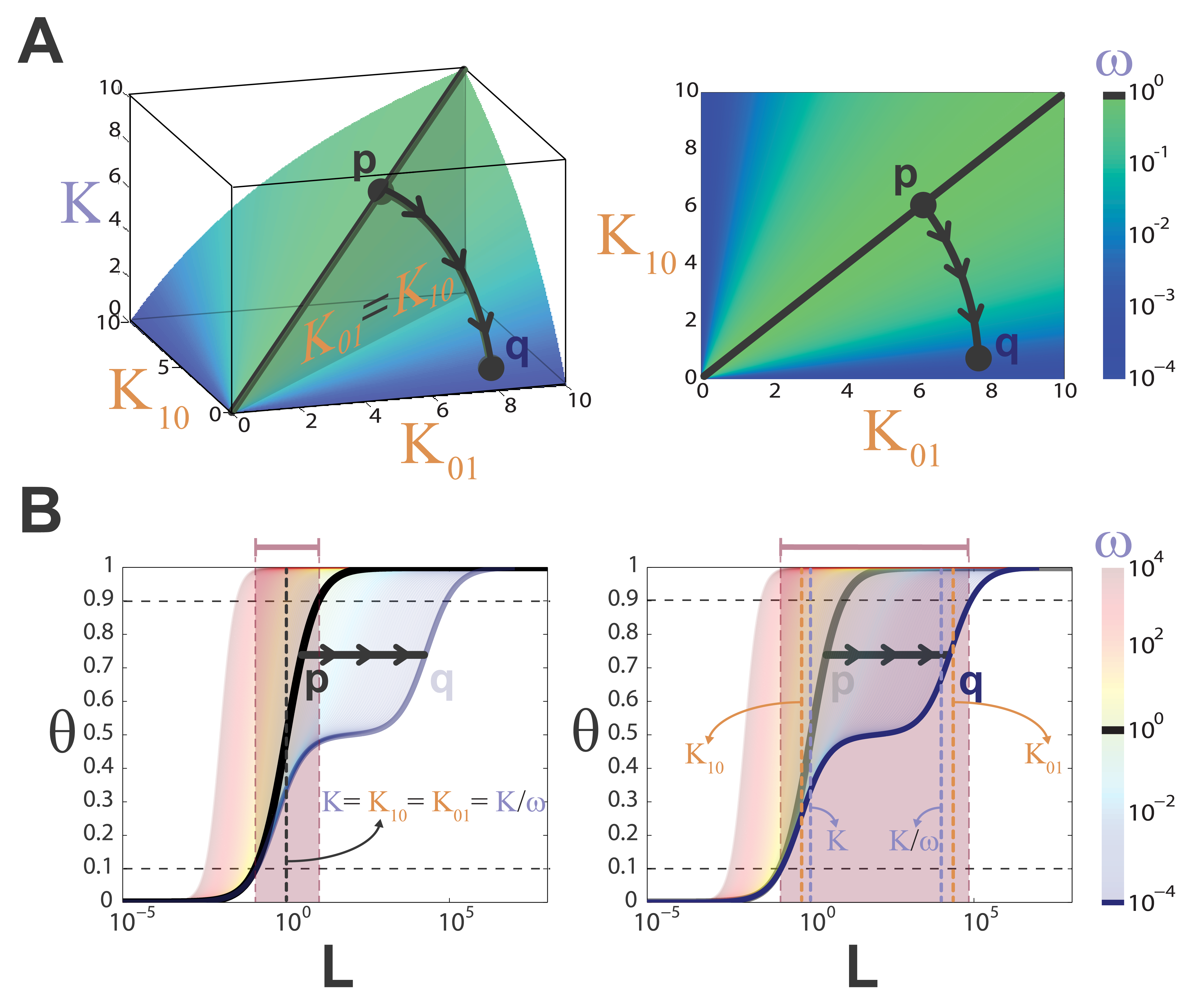
**

**Fig A: Non-identifiability manifold obtained from Eqs. (3) and (4) in the main text.** (A). (left panel) The manifold is represented in the 3 ratios ($K$, $K_{10}$ and $K_{01}$) space, with $\omega$ in color scale. One point in this plot represents one set of$\left( K,\omega\right)$ from **NC** and ($K_{10}$, $K_{01}$) from **IB** which have the same dose-response curves in equilibrium. Each point represents infinite different sets of **NC** or **IB** parameters, as binding ($k$) and unbinding ($l$) rates can change while leaving its ratio unchanged. The vertical gray plane represents the region where $K_{10}{=K}_{01}$ and the black straight line in the intersection between the plane and the manifold is both where $\omega=1$ and the identity $K=K_{10}{=K}_{01}$. Notice that for a given set of $\left( K,\omega\right)$, there are two possible sets of ($K_{10}$, $K_{01}$) which have the same dose-response curves, but these sets are those with interchanged ratios $K_{10}$ ↔ $K_{01}$. The straight black line is a symmetry axis of the manifold and represents both model’s symmetry in swapping receptor binding sites. (right panel) Same as the left panel but looked from above. Notice that $\omega$ defines a proportional relation between $K_{10}$ and $K_{01}$ (the same color curves are straight lines crossing the origin, see section 2a). The black dots **p** and **q** and the trajectory from **p** to **q** are marked as an example to explain the non-identifiability conditions of Eqs. (3) and (4), going from no cooperativity $\left( \omega=1 \right)$ to a very negative one $\left( \omega\approx0 \right)$. (B). Both model’s equilibrium dose-response curves, as shown in Fig 1C in the main text, but with **p** (left panel) and **q** (right panel) curves highlighted and its ratios marked with vertical dashed lines. Vertical dashed pink lines are the EC_10_ and EC_90_ for both curves, and the $\mathbf{DynR}$ region is colored in pink too, with the value of $\mathbf{DynR}$ indicated over the planel. In **NC**, going from **p** to **q** means decreasing $\omega$, increasing the middle region where $\boldsymbol{\theta}=\frac{1}{2}$, as more ligand concentration is needed for the second binding to occur. This increases the $\mathbf{DynR}$. In **IB**, notice that the same middle region appears as curves are the same but due to both sites different affinities. Here, in the middle region, the most affine site (10) is already saturated but the second site (01) is less affine and needs more ligand to respond, increasing $\mathbf{DynR}$.

## 1.2 Features of the $\mathbf{DynR}$ versus time curve.

### 1.2a. The cooperativity factor $\boldsymbol{\omega}$ (in NC) defines the relation between $\mathbf{K}_{\mathbf{01}}$ and $\mathbf{K}_{\mathbf{10}}$ (in IB).

Dividing the non-identifiability conditions (Eqs. (3) and (4)), we get the following expression for $\omega$:

$\omega=\frac{4K_{10}K_{01}}{\left( K_{10}+K_{01} \right)^{2}}$ (S1)

which gives the following quadratic expression for the ratio between $K_{01}$ and $K_{10}$:

$\left( \frac{K_{10}}{K_{01}} \right)^{2}\omega+\frac{K_{10}}{K_{01}}\left( 2\omega-4 \right)+\omega=0$

The two solutions are given by:

$\frac{K_{10}}{K_{01}}=\frac{2-\omega\pm2\sqrt{1-\omega}}{\omega}$

These two solutions exist only for $\omega<1$, recovering the fact that non-identifiability occurs only for negative cooperativity. Notice that $\omega$ defines two possible proportional relations between the ratios $K_{01}$ and $K_{10}$, as shown in Fig AB. The freedom in choosing one or the other solution represents the symmetry in swapping binding sites. If we choose one solution and then swap sites to get the inverse relation $\frac{K_{01}}{K_{10}}$ , from the previous expression, it results in the following expression:

$\frac{K_{01}}{K_{10}}=\frac{\omega}{2-\omega\pm2\sqrt{1-\omega}}= \frac{\omega}{2-\omega\pm2\sqrt{1-\omega}} \frac{2-\omega\mp2\sqrt{1-\omega}}{2-\omega\mp2\sqrt{1-\omega}}=\frac{2\omega-\omega^{2}\mp2\omega\sqrt{1-\omega}}{\left( 2-\omega\right)^{2}-4\left( 1-\omega\right)}=\frac{2-\omega\mp2\sqrt{1-\omega}}{\omega}$

Notice the last right-hand side is just the other solution. This means that one solution is just the inverse of the other, i.e., given a value of $\omega,$ the ratio of the non-identifiable pair ($K_{10}$,$K_{01}$) can be chosen to be $\frac{2-\omega+2\sqrt{1-\omega}}{\omega}$ and its inverse $\frac{2-\omega-2\sqrt{1-\omega}}{\omega}$ or backwards, which means to interchange binding sites. Multiplying Eq. (S1) by $K_{10}K_{01}$, we get a simpler expression to see the symmetry in swapping sites:

$\omega=\frac{4}{\frac{K_{10}}{K_{01}}+\frac{K_{01}}{K_{10}}+2}$ (S2)

### 1.2b Equilibrium NC dynamic range decreases with $\boldsymbol{\omega}$.

From Eq. (2) we can analytically calculate $EC_{90}$ and $EC_{10}$for $t\to\infty$ and then the $\mathbf{DynR}$ with Eq. (8).

$0.9=\frac{EC_{90}K+\omega EC_{90}^{2}}{K^{2}+2EC_{90}K+\omega EC_{90}^{2}}$ $0.1=\frac{EC_{10}K+\omega EC_{10}^{2}}{K^{2}+2EC_{10}K+\omega EC_{10}^{2}}$

Solving the quadratic expressions:

$EC_{90}\left( t\to\infty\right)=K\frac{0.8\pm\sqrt{{0.8}^{2}+4\omega0.09}}{2\omega0.1}$ $EC_{10}\left( t\to\infty\right)=K\frac{-0.8\pm\sqrt{{0.8}^{2}+4\omega0.09}}{2\omega0.9}$

And then we can obtain an expression for $\mathbf{DynR}\left( \omega\right)$, which is independent of any other parameter.

$\mathbf{DynR}\left( t\to\infty\right)=\ln\left( 9 \right)+\ln\left( \frac{1+\sqrt{1+\left( \frac{3}{4} \right)^{2}\omega}}{-1+\sqrt{1+\left( \frac{3}{4} \right)^{2}\omega}} \right)$ (S3)

Notice that this is a monotonic decreasing function that diverges for $\omega\to0$ (the second binding can never happen), passes trough $\ln\left( 81 \right)$ in $\omega=1$ and tends to $\frac{\ln\left( 81 \right)}{2}$ when $\omega\to\infty$, which is consistent with a Hill coefficient of 2 because of the 2 binding sites. This can be seen in Fig AB and explains why the non-identifiability problem only happens for negative cooperativity. For positive cooperativity, $\mathbf{DynR}<ln(81)$ and this is unachievable for **IB**.

### 1.2c Equilibrium dynamic range comparison.

It is possible to get an expression of $\mathbf{DynR}\left( t\to\infty\right)$ for **IB**, as a function of $K_{10}$ and $K_{01}$. As $\mathbf{DynR}$ comes from dose-response curves, we can replace $\omega$ in Eq. (S3) using Eq. (S2), resulting in the following expression:

$\mathbf{DynR}\left( t\to\infty\right)=\ln\left( 9 \right)+\ln\left( \frac{1+\sqrt{1+\frac{9/4}{K_{10}/K_{01} +K_{01}/K_{10}+2}}}{-1+\sqrt{1+\frac{9/4}{K_{10}/K_{01} +K_{01}/K_{10}+2}}} \right)$ (S4)

Eqs. (S3) and (S4) are shown in Fig B where it can be seen that is impossible to reach values under $\ln\left( 81 \right)$ from the **IB** model and this makes the non-identifiability problem to exist only for negative cooperativity. The $\mathbf{DynR}$ shown corresponds to the equilibrium dose-response curves in Fig 1 in the main text and Fig A.

**
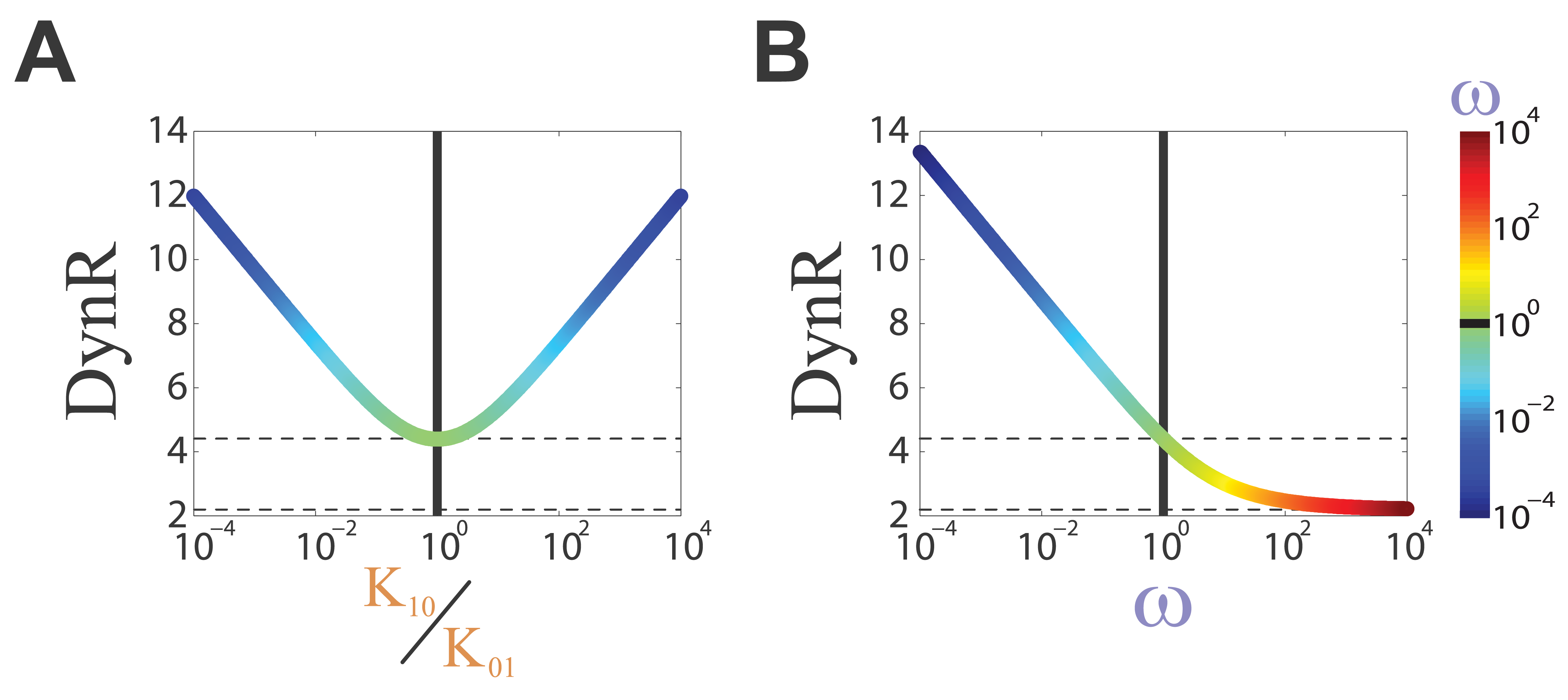
**

**Fig B: Equilibrium** $\mathbf{DynR}$**.** Both panels show $\omega$ (left) and effective $\omega$ (right) in color scale and $\omega=1$ with a black vertical line. Dashed horizontal lines are $\ln\left( 81 \right)$ and $\ln\left( 81 \right)/2$. (A)**.** $\mathbf{Dyn}\mathbf{R}\left( t\to\infty\right)$ **versus IB** **ratios relation**. The curve is symmetrical for swapping sites, as **IB** is, and both minimums of $\omega$ and $\mathbf{DynR}$ are located where the ratios are equal. (B)**.** $\mathbf{DR}\left( t\boldsymbol{\to\infty} \right)$ **versus** $\boldsymbol{\omega}$. $\mathbf{Dyn}\mathbf{R}$ is always decreasing with $\omega$ and reaches values under $\ln\left( 81 \right)$ for $\omega>1$ (positive cooperativity).

### 1.2d Early NC dynamic range is controlled by $\boldsymbol{\omega}$.

The control of $\mathbf{DynR}\left( t\to0 \right)$ by $\omega$ can be analytically obtained. Taking the differential equations that describe the evolution of the **NC** model, Eqs. (10), we can make an ‘early times approximation’, which means considering $t\to0$ and $K\ll L$ or take a high ligand concentration to look at the binding dynamics, before any appreciable unbinding can happen ($K\ll L$ implies $l\ll kL$, or the binding rates are much higher than the unbinding ones). This approximation simplifies the system as follows, where a dimensionless time was defined as $\tau=k\mathrm{Lt}$:

$\frac{d}{d\tau}\binom{R^{10}+R^{01}}{R^{11}}= \left( \begin{matrix} -(2+\omega) & -2 \\ \omega& 0 \end{matrix} \right)\binom{R^{10}+R^{01}}{R^{11}}+R_{0}\binom{2}{0}$

Solving this system leads to

$\boldsymbol{\theta}\left( \tau\to0 \right)=\frac{1}{2-\omega}\left[ \left( \omega-1 \right)e^{-2\tau}-e^{-\omega\tau} \right]+1$

Where $-2$ and $-\omega$ are the eigenvalues that corresponds to the first bindings to both sites and the second and slower bindings, respectively. The relation between both time scales is just $\frac{\omega}{2}$.

We can now calculate EC_90_, EC_10_ and $\mathbf{DynR}$, where $\tau_{90/10}=kEC_{90/10}t$.

$0.9=\frac{1}{2-\omega}\left[ \left( \omega-1 \right)e^{-2\tau_{90}}-e^{-\omega\tau_{90}} \right]+1$ $0.1=\frac{1}{2-\omega}\left[ \left( \omega-1 \right)e^{-2\tau_{10}}-e^{-\omega\tau_{10}} \right]+1$ (S5)

Even though we have reached to two transcendental equations, it is possible to see that both $\tau_{90/10}$ are $\omega$-dependent. Going back to the definition of $\tau$, we have:

$EC_{90/10}\left( t\to0 \right)=\frac{\tau_{90/10}\left( \omega\right)}{kt}$

And then

$\mathbf{DynR}\left( t\to0 \right)=\ln\left( \frac{\tau_{90}\left( \omega\right)}{\tau_{10}\left( \omega\right)} \right)$ (S6)

We demonstrated that $\mathbf{DynR}\left( t\to0 \right)$ tends to a constant in time and it can only depend on $\omega$, as shown numerically in Fig 3B and Fig 3C in the main text and in Fig BB. Both $\tau_{90/10}\left( \omega\right)$ functions can be numerically obtained from Eq. (S5) and replacing its values in Eq. (S6) confirm the curve $\mathbf{DynR}\left( t\to0 \right)$ vs $\omega$ is the same as the one shown in Fig 3C in the main text, which comes from integrating **NC**.

### 1.2e Early IB dynamic range is controlled by $\boldsymbol{k}_{\mathbf{10}}$/$\boldsymbol{k}_{\mathbf{01}}$.

The same ‘early times approximation’ can be applied to **IB** to get the following expression:

$$\boldsymbol{\theta}\left( L\to\infty,t\to0 \right)=\frac{1}{2}\left( 1-e^{- k_{10}\mathrm{Lt}}+1-e^{- k_{01}\mathrm{Lt}} \right)$$

We can see that now the eigenvalues are $-k_{10}L$ and $-k_{01}L$ and there are two different and independent time-scales, and the relation between them is $k_{10}$/$k_{01}$. This depends on two parameters (instead of only one like in **NC**) and is the reason of both model’s difference in $\mathbf{DynR}\left( t\to0 \right)$ that is the basis of Checkpoint 2 and is shown in Fig 3A, Fig 3B and Fig 3C in the main text and in Fig BA and Fig BB. This difference also leads to three different regimes for $\mathbf{DynR}\left( t \right)$ curves, explained in the following subsection.

We can calculate again EC_90_, EC_10_ and $\mathbf{DynR}$ as before,

$0.9=\frac{1}{2}\left( 1-e^{- k_{10}EC_{90}t}+1-e^{- k_{01}EC_{90}t} \right)$ $0.1=\frac{1}{2}\left( 1-e^{- k_{10}EC_{10}t}+1-e^{- k_{01}EC_{10}t} \right)$

$e^{- k_{10}EC_{90}t}+e^{- k_{01}EC_{90}t}=0.2$ $e^{- k_{10}EC_{10}t}+e^{- k_{01}EC_{10}t}=1.8$

We reach again to two transcendental equations. We have numerically solved them, and the result is consistent with the numerically obtained function for $\mathbf{DynR}\left( t\to0 \right)$ vs $k_{10}$/$k_{01}$ shown in Fig 3C in the main text and Fig CB which comes from integrating **IB**.

### 1.2f IB dynamic range regimes are controlled by $\boldsymbol{k}_{\mathbf{10}}$/$\boldsymbol{k}_{\mathbf{01}}$.

As shown in the previous subsection, for early times and when binding rates are much higher than unbinding rates, these last ones can be neglected. This leads to two different time-scales in **IB** early times, controlled by $k_{10}$ and $k_{01}$. And so, the $\mathbf{DynR}\left( t\to0 \right)$ is then controlled by the relation between these two time-scales, as discussed in the previous subsection. However, equilibrium$\mathbf{DynR}$ depends on the equilibrium ratios, where unbinding rates cannot be neglected. This implies that, from the four independent parameters in the **IB** model, there are just two that control $\mathbf{DynR}\left( t\to0 \right)$ ($k_{10}$ and $k_{01}$) and, given these two, the other two ($l_{10}$ and $l_{01}$) can be used to choose any equilibrium value for $\mathbf{DynR}$. In Fig C, three possible regimes are shown (dose-response curves and temporal curves are included for one example on each regime). Each example has a very different $k_{10}$/$k_{01}$ relation $\left( \frac{k_{10}}{k_{01}}\gg1,\frac{k_{10}}{k_{01}}=1 ,\frac{k_{10}}{k_{01}}\ll1 \right)$, but the unbinding rates were chosen to give the same equilibrium ratios. The parameters values for each example are listed in Table A.

**
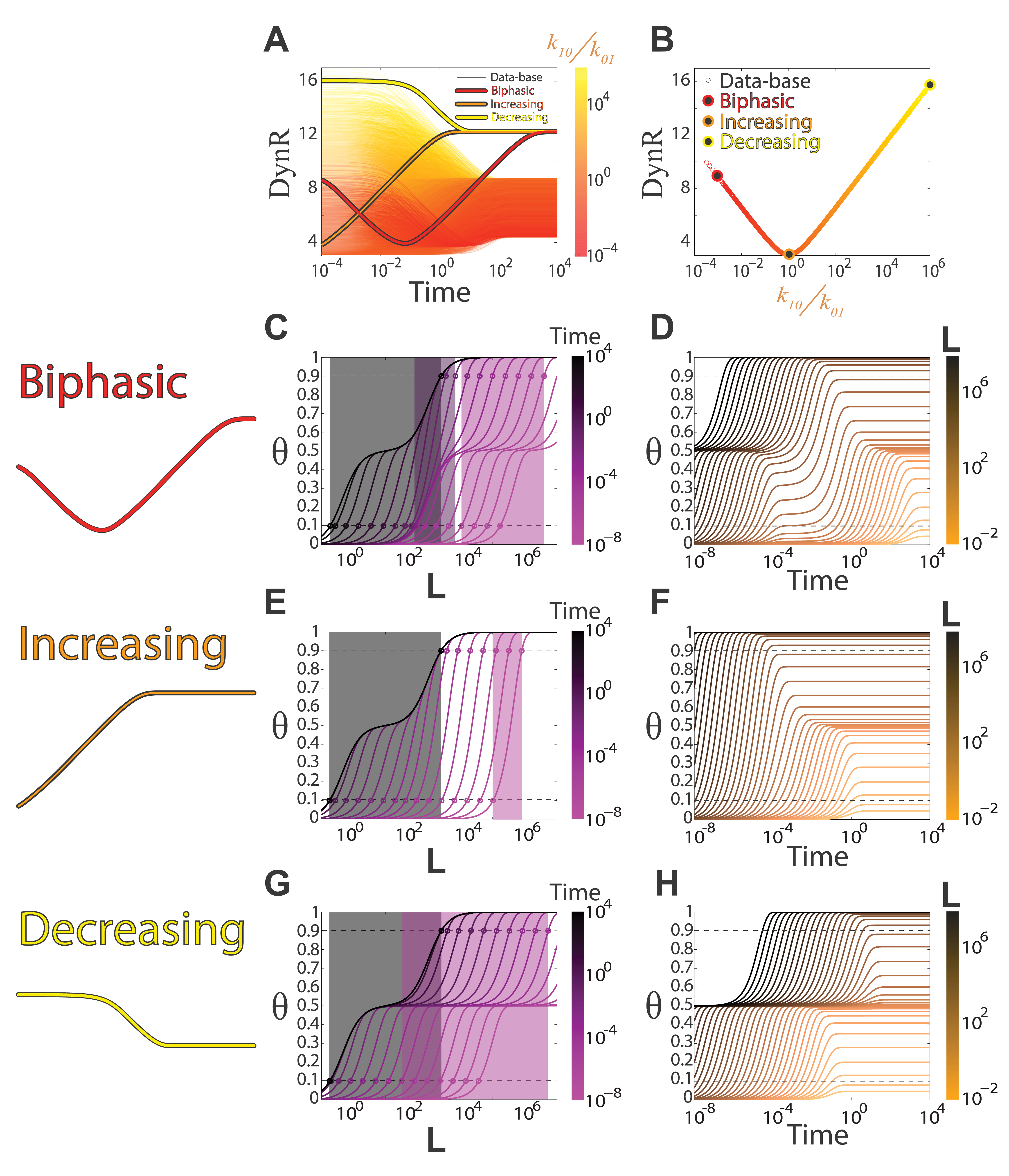
**

**Fig C:** $\mathbf{DynR}$ **versus time in the IB** **model, three different behaviors.** (A)**.** $\mathbf{DynR}$ versus time for the **IB** database, with $k_{10}$/$k_{01}$ indicated in a colorscale. At $t\to0$ all the curves are ordered by $k_{10}$/$k_{01}$ and this relation defines three different regimes: biphasic, increasing and decreasing curves, for $k_{10}\ll k_{01}$, $k_{10}\cong k_{01}$ and $k_{10}\gg k_{01}$ respectively. These regimes are represented with three example curves that have been chosen from its $k_{10}$/$k_{01}$value, low for the increasing curve, middle for the biphasic one and high for the decreasing curve. These values are the points marked in panel (B). (B)**.** $\mathbf{DynR}\left( t\to0 \right)$ versus $k_{10}$/$k_{01}$. $\mathbf{DynR}\left( t\to0 \right)$is a continuous and non-monotonic function of this relationship of parameters. Three points are marked as examples of three different regimes. (C) and (D) biphasic behavior; (E) and (F) increasing behavior; (G) and **(**H) decreasing behavior. The examples included in (C-H) are those indicated in panels (A) and (B). Left panels show dose-response curves for different times (time in a color scale) and right panels show temporal curves for different doses (dose in a color scale). **Biphasic**. The site 10 was chosen as the most affine site ($K_{10}<K_{01}$) with no loss of generality. Then, $k_{10}\ll k_{01}$ means that the most affine site is also the slowest for earlier times (when unbinding rates are neglected), where $\mathbf{DynR}$ is large because it needs much more ligand concentration to make the slow binding to site 10 to occur (light pink region). In equilibrium, $\mathbf{DynR}$ is larger too, but because unbinding rates cannot be neglected and the relation between $K_{10}$ (lower, most affine) and $K_{01}$ (higher, less affine) needs to be compensated by a larger ligand concentration. For intermediate times, $\mathbf{DynR}$ gets shorter because the concentration of ligand needed to bind to the slower 10 site balance the one needed to bind to the less affine site 01. **Increasing**. As $k_{10}=k_{01}$, both sites have similar binding timescales for early times and both binding dynamics are similar too, resulting in a shorter $\mathbf{DynR}$ (light pink region). However, in equilibrium, the different unbinding rates matter and, as before, the relation between the same $K_{10}$ and $K_{01}$ needs to be compensated by a larger ligand concentration, resulting in a larger $\mathbf{DynR}$ (dark region). **Decreasing**. In this example, $k_{10}\gg k_{01}$, which means the most affine site is also the fastest one for earlier times. The site 10 does not need much ligand for the binding to occur, even for early times. On the opposite, the site 01 needs a much higher concentration for these times as it is slower, making a very large $\mathbf{DynR}$, the slower 01, the larger $\mathbf{DynR}\left( t\to0 \right)$. Then, in equilibrium, unbinding rates matter and, as $l_{10}\gg l_{01}$to get the same $K_{10}$ and $K_{01}$ as before, there are more unbindings in the 10 site (most affine) than in the 01 site, giving a higher ligand concentration to make the most affine binding to occur. This results in a shorter $\mathbf{DynR}$.

# 2. Testing the algorithm: Simulated and experimental data

## 2.1 Thresholds in the algorithm.

Every step or checkpoint in the algorithm described in this paper needs the definition of a threshold. Based on each threshold, the decision of continuing or not is made. The last checkpoint is the exception because, there, the decision (either the identity of the data is **IB** or **NC**) must be made.

In Fig D we include the complete databases **IB** and **NC** colored according to the value of $\omega$ and $l$, respectively (in the main text we used some filters in these two databases for clarity purposes).

In S1E Fig we plot the predicted values of $\mathbf{DynR(}t\to0\mathbf{)}$ (Fig EA and Fig EC) and t_ip_ (Fig EE) versus the directly measured ones for the simulated datasets. The predictions come from the functions according to Fig 3 in the main text and based on the values of $\omega$, $k_{10}$/$k_{01}$ and $l$ obtained from Steps 1-3 of the TC algorithm. By analyzing those plots, we define the thresholds for Checkpoints 2 and 3 in the algorithm, as explained in the figure. Furthermore, in what follows we explain the optimization that we performed to obtain the value of each threshold needed in the algorithm. Each one was calculated applying the algorithm with simulated data with a fixed level of stochasticity, i.e. R_0_=1000, and for 100 sets of each model.

**
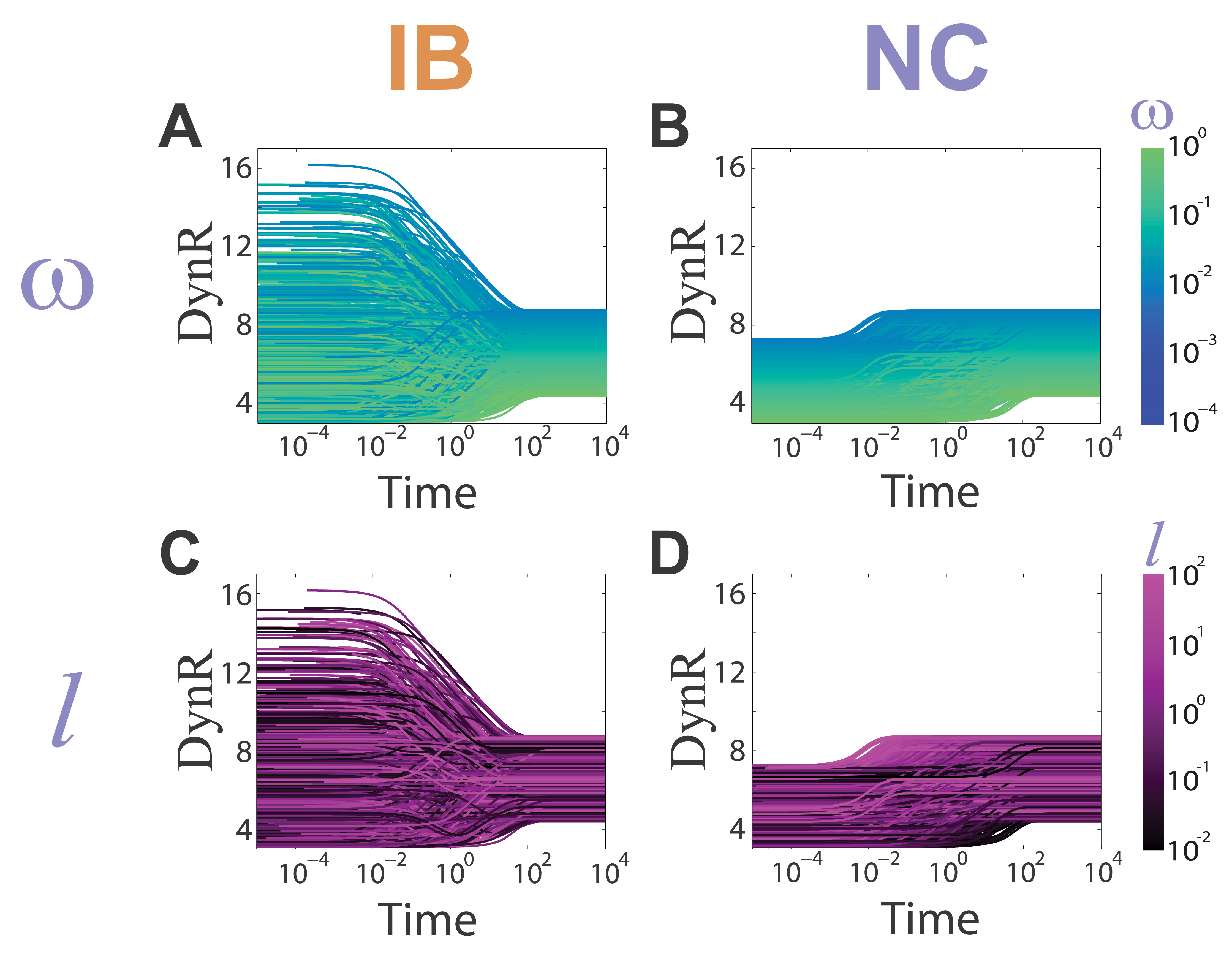
**

**Fig D: The dose-response curve evolves in time differently for IB** **and NC models, full databases.** In Fig 3 in the main text, we show plots of $\mathbf{DynR}$ versus time computed from 10000 parameter sets chosen randomly in the non-identifiability manifold. In that figure and for clarity purposes, panels (A) and (B) contain only curves with 0.9 < $l$ < 1.1 (207 curves), and panels (G) and (H) contain only curves with 0.010 < $\omega$ < 0.011 (218 curves). In this figure we include the corresponding full databases.

**
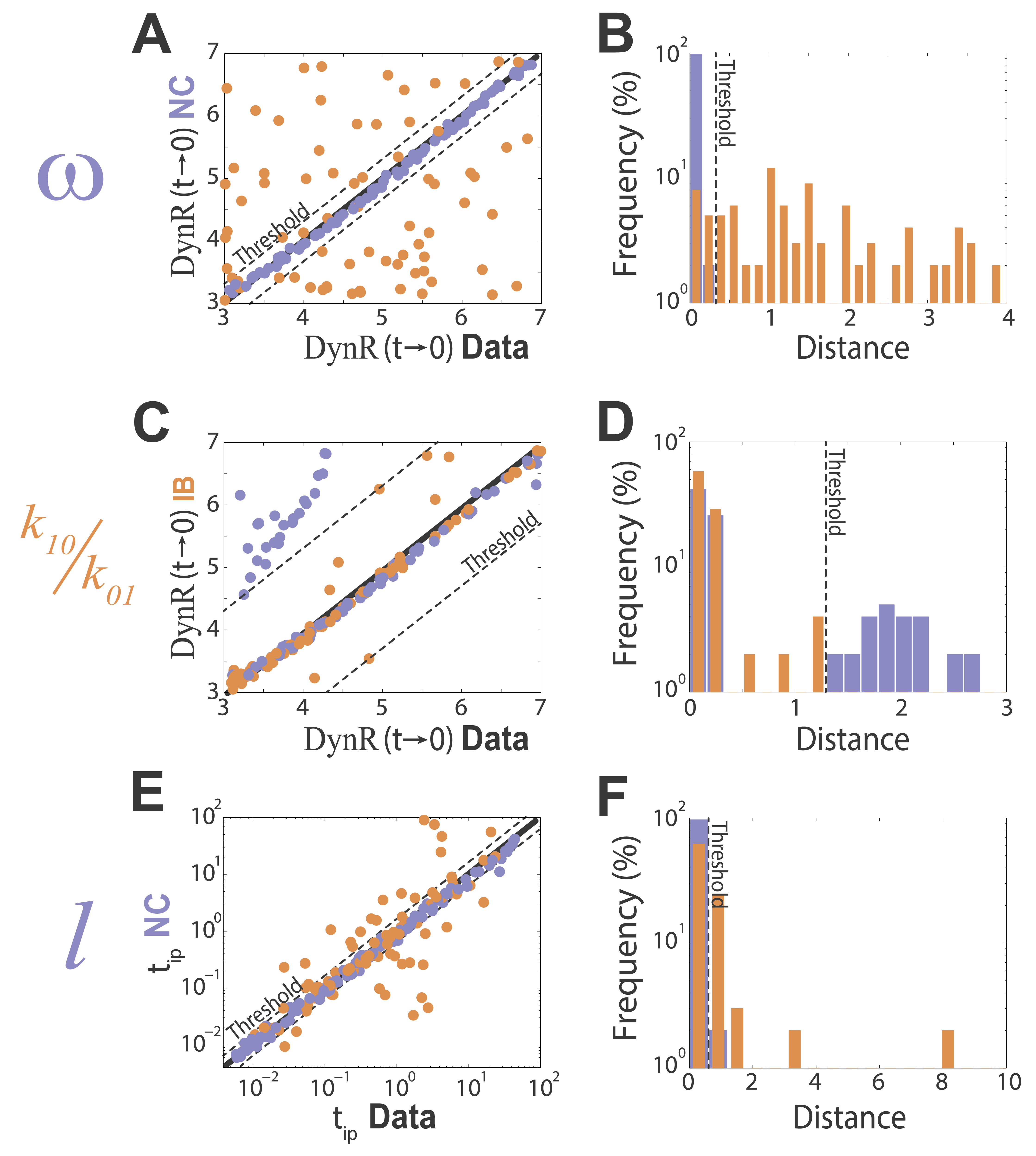
**

**Fig E: Thresholds definitions for Checkpoints 2 and 3.** 100 **NC** datasets and 100 **IB** datasets with 1000 receptors each of them was simulated. $\mathbf{DynR}\left( t\to0 \right)$ and t_ip_ were computed from the data for those 200 cases and are compared with the predicted values expected from functions according to Fig 3 in the main text and based on the values of $\omega$, $k_{10}$/$k_{01}$ and$l$ obtained from Steps 1-3 of the TC algorithm. Predicted $\mathbf{DynR}\left( t\to0 \right)$ (A) and (C) and predicted t_ip_ (E) are plotted versus the calculated ones, indigo/brown dots for **NC**/**IB** respectively. The black line represents the identity function. In (A), indigo dots (**NC**) lie over the identity while brown dots are everywhere. In (B), mostly brown dots (**IB**) lie over the identity. In (C), mostly indigo dots lie over the identity. Small deviations are due to errors in the value of the fitted parameters and can be attributed to data stochasticity. The black dashed lines over panels (A), (B), and (C) are the thresholds for Checkpoints 2 and 3 calculated based on panels (B), (D), and (F), respectively. In those panels we plot the distributions of the distances to the identity for **NC** (indigo) and **IB** (brown) datasets. Comparing both distributions, it can be defined a threshold: if the measured value of $\mathbf{DynR}\left( t\to0 \right)$ or t_ip_ distance is above that threshold, then the identity of the data is clear, if it is below, it is not possible to decide.

Step 3: the value of ***C***. **IB** and **NC** models have different number of parameters, **IB** has 4 parameters ($k_{10}$, $k_{01}$, $l_{10}$, $l_{01}$) and **NC** has 3 ($k,l,\omega$). We applied the F-test (1), using *fcdf* Matlab function (F cumulative distribution function) to penalize the fit on **IB** because of having more parameters. The F-test takes both fits residuals mean-square distances and gives the probability on **IB** (the one with more parameters) to fit better than **NC**, taking on account the different number of parameters. This probability is a number between 0 and 1 that we called ***C*** (Fig FA). ***C*** = 0 means absolute certainty that the data comes from **NC** model, ***C*** = 1 is absolute certainty that the data comes from **IB** model, and ***C*** = ½ means absolute uncertainty. The thresholds we used resulted from the optimization we describe next and are **1/3 and 2/3**. If ***C*** < 1/3 the protocol defines for **NC**, if ***C*** > 2/3, for **IB** and if ***C*** is in between, the protocol continues to the following steps. 1/3 and 2/3 were selected to have the same distance to 1/2, being this distance the only parameter to optimize. We then took the minimum distance that made no mistakes in deciding the identity of the data (Fig FB). For lower distances, the algorithm decides this identity based on two more similar fittings, increasing the chances of error.

Checkpoint 1: detecting non-increasing $\mathbf{DynR(}t\mathbf{)}$ curves. To define if the $\mathbf{DynR(}t\mathbf{)}$ curve is decreasing or biphasic, considering the stochastic fluctuations involved, we first made an estimation of $\mathbf{DynR}\left( t\to0 \right)$ considering the first 100 data points of the $\mathbf{DynR(}t\mathbf{)}$ curve. From the corresponding curves for the deterministic examples we analyzed, we know that the first 100 data points belong to the constant region of the curves. Then, we calculated the threshold as Eq. (S7), where $mean$ and $sd$ are the mean and standard deviation of the $\mathbf{DynR}\left( t\to0 \right)$ distribution and $X$ is the parameter to optimize, i.e. the threshold.

$threshold=mean\left( \mathbf{DynR}\left( t\to0 \right) \right)-X*sd\left( \mathbf{DynR}\left( t\to0 \right) \right)$ (S7)

The optimization was as before, choosing the minimum value of $X$ that has no incorrect definitions, which is **2.7**. For lower values, there are stochastic fluctuations that undergo the threshold for **NC** data examples, resulting in an incorrect definition ($\mathbf{DynR(}t\mathbf{)}$ is always increasing in **NC**). For higher values, the chances of making an incorrect definition in this checkpoint decrease, but more **IB** examples that would be correctly defined end up going through this step.

Checkpoint 2. The two thresholds for the distances between $\mathbf{DynR}\left( t\to0 \right)\mathbf{Data}$ and predicted $\mathbf{DynR}\left( t\to0 \right) \mathbf{NC}$ according to the value of $\omega$ or $k_{10}/k_{01}$, are shown in Fig FA-D. The value of $\mathbf{DynR}\left( t\to0 \right)\mathbf{Data}$ was calculated taking the mean of the first 100 data points as in the previous threshold. The optimization for both was done as before, choosing the maximum value that has no incorrect definitions. In the case of $\omega$, for lower values there are stochastic fluctuations beyond the threshold for examples coming from **NC** model, resulting in an incorrect **IB** definition. For higher values, the chances of making an incorrect definition in this checkpoint decrease, but more **IB** examples that would be correctly defined end up going through this step. In the case of $k_{10}/k_{01}$, for lower values there are stochastic fluctuations beyond the threshold for examples coming from **IB** model, resulting in an incorrect **NC** definition. For higher values, the chances of making an incorrect definition in this checkpoint decrease, but more **NC** examples that would be correctly defined end up going through this step.

Checkpoint 3. The last threshold is for $t_{\mathrm{ip}}$ to predict its value by the value of the rate $l$, shown in Fig FE and Fig FF. The estimation of $t_{\mathrm{ip}}$ needs the local derivative of $\mathbf{DynR}\left( t \right)$, and as the data has stochastic fluctuations, it is calculated fitting all the intervals of 100 neighboring points from the $\mathbf{DynR}\left( t \right)$ curve to a linear function in log_10_ scale, and getting the slope as the derivative of the middle point of the time interval. This derivative reaches its maximum at $t_{\mathrm{ip}}$ and is independent of any parameter (Fig F), so we numerically calculated this maximum and defined $t_{\mathrm{ip}}$ as the time when the slope reaches the maximum. This has an advantage over calculating the second derivative or looking at the peak of the first derivative, which can be very noisy when the data comes from stochastic simulations. As this is the last checkpoint of the protocol, it always makes a definition, so it was optimized differently from previous steps. The threshold was chosen to just maximize the correct definitions, which is equivalent to minimize the incorrect ones, given that there is always a definition.

**
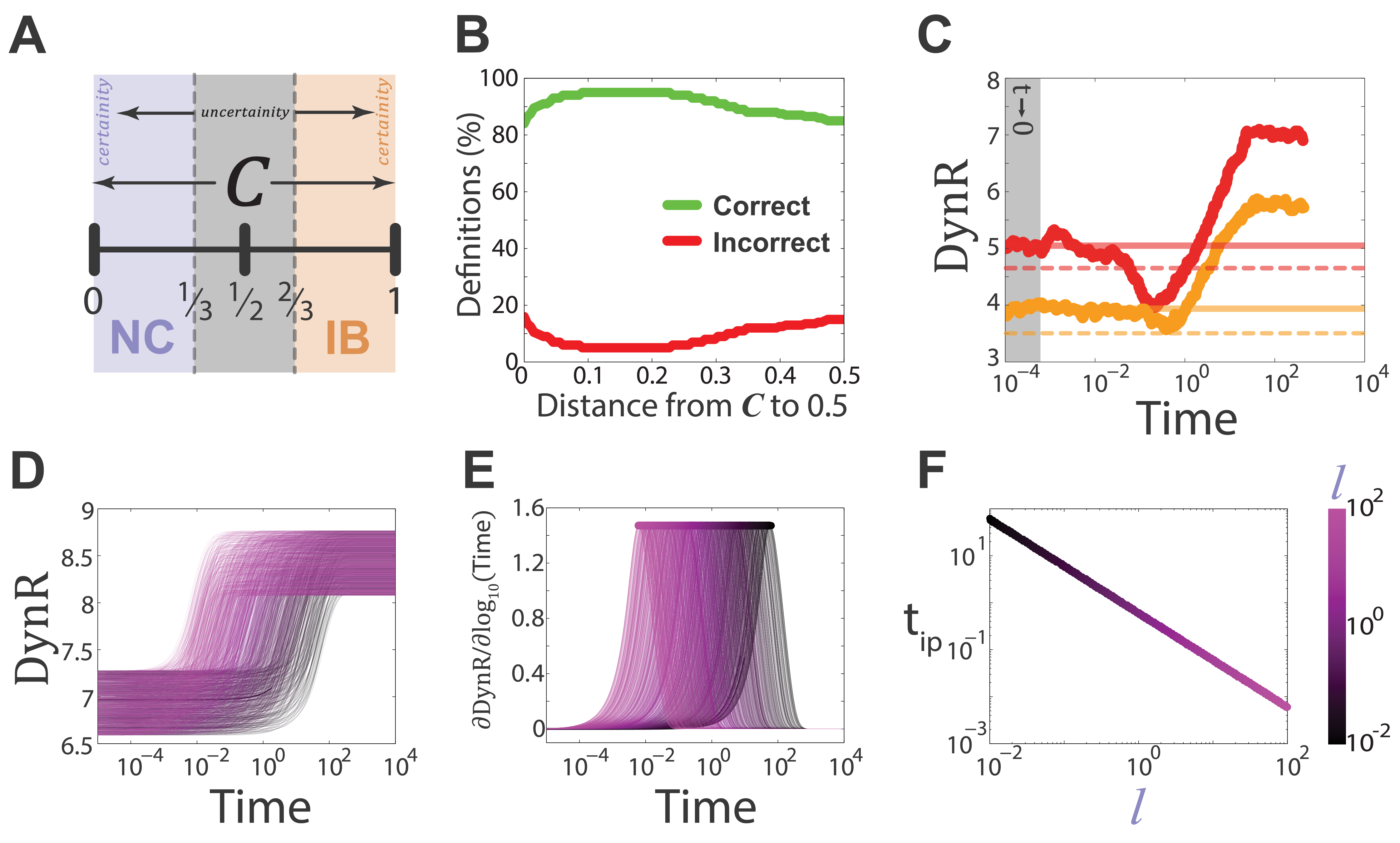
**

**Fig F: Thresholds in the algorithm. Top panels.** (A)**, threshold for Step 3.** Definition of the value of ***C***, ½ is absolute uncertainty and both extreme values 0 and 1 represent absolute certainty that the identity of the model is **NC** or **IB**, respectively. The thresholds were chosen as 1/3 and 2/3, between these values the protocol does not define and continues to Checkpoint 1. (B)**, TC+DR algorithm efficiency dependence with *C***. Correct and Incorrect definitions versus distance from ***C*** to ½. Results from the test with stochastic data using 1000 total receptors. The chosen value of ***C*** arises from that producing maximum correct definitions of the algorithm and minimum incorrect ones. (C)**, threshold for Checkpoint 1**. Two $\mathbf{DynR}$ versus time curves obtained from simulated stochastic datasets, one from the biphasic regime (red) and the other from the increasing regime (orange). Thresholds, as defined in Eq. (S7), are shown for each example in dashed red lines while mean values of the first 100 curve points (highlighted gray $t\to0$ region) are in filled lines. Notice that the biphasic curve goes below its threshold and the increasing curve does not. DR protocol decides the biphasic curve is from **IB** but in the increasing curve example, it continues to Checkpoint 2.

**Bottom panels. t_ip_ dependency with parameter** $\boldsymbol{l}$. (D). $\mathbf{DynR}\left( t \right)$ for the **NC** database, 1500 curves filtered with $\omega$ being between 0.001 and 0.02 for clarity. Curves have the same shape and are ordered by the value of $l$, from right to left as $l$ increases. (E). Logarithmic time derivative of the $\mathbf{DynR}\left( t \right)$ curves shown in (D). The maximum of each curve is the inflexion point, marked with a circle. Notice that all maximums are equal, $\approx1.5$, and this value is independent of any parameter. (F). t_ip_ for the inflexion points marked in (E) in terms of $l$ represented both in the x-axis and in color-scale. t_ip_ versus $l$ is a continuous function. This function is the one shown in Fig 3I in the main text and is the one used for the last checkpoint of the protocol.

## 2.2 The algorithm applied to experimental data.

Fig G shows the TC+DR protocol applied to both experimental sets of **IB** and **NC**, step by step. The decision is made in Checkpoint 2 ($\omega$) for the **IB** set, because $\mathbf{DynR}\left( t \right)$ curve is below the predicted **NC** value. For **NC**, the protocol reaches the final Checkpoint 3, where the maximum derivative of the curve corresponds to the one predicted by **NC** ($\approx1.5$) and t_ip_ corresponds to the value predicted by $l$ parameter.

**
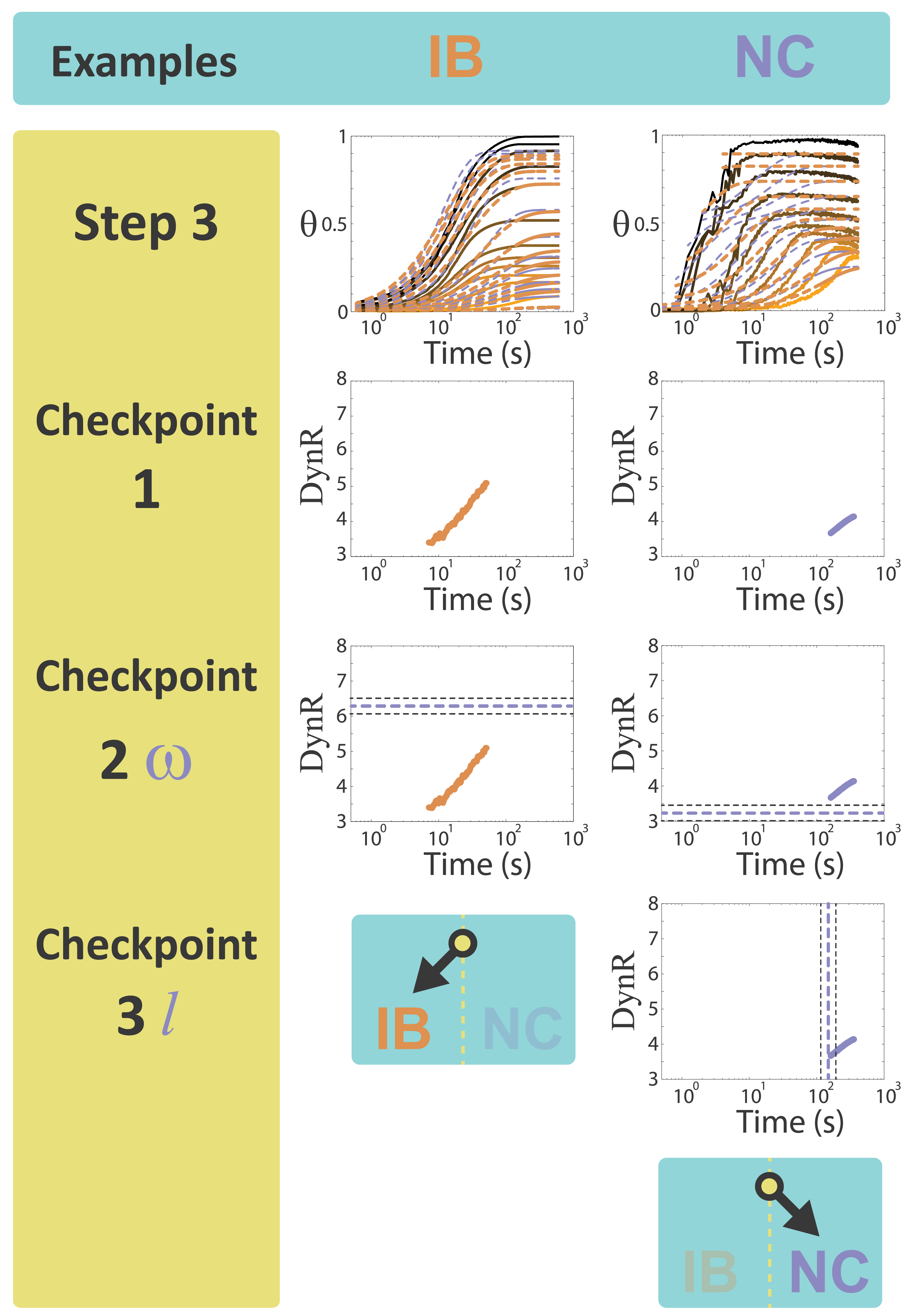
**

**Fig G: TC + DR algorithm applied to experimental data**. First column contains the **IB** example, second column the **NC** one. First row, input data: $\boldsymbol{\theta}$ vs. time for different values of $L$. The rows follow the same structure as Fig 5 in the main text (except for Checkpoint 2 $k_{10}$/$k_{01}$, which is not used for the experimental data). **IB** **data**, the algorithm cannot decide in Step 3, so the $\mathbf{DynR}$ analysis is performed. Since all the $\mathbf{DynR}$ values are below the $\mathbf{DynR}\left( t\to0 \right)$ predicted by the **NC** model and $\mathbf{DynR}\left( t\to0 \right)$ in this model should be an increasing function of time, it is concluded that the identity of the data is **IB**. **NC data**, the algorithm cannot decide in Step 3 nor in Checkpoints 1 or 2, but according to the value of t_ip_, it is concluded that the identity of the data is **NC**. Even though it is not possible to compute t_ip_ with this data, we computed $\mathbf{DynR}\left( t \right)$ maximum derivative, as described in Methods section.

Table 1 in the main text shows the equilibrium ratios obtained for the four experimental data cases described in Fig 7 in the main text, fitting the equilibrium dose-response curve from each one with both model’s predictions (Eq. (1) for $K_{10}$ and $K_{01}$ and Eq. (2) for $K$ and $\omega$). These ratios are compared with $K_{1}$ and $K_{2}$, which were reported in each paper fitting data with a two-site model. In the first example, where both sites are equal, all ratios are similar.

In the experimental papers cited in this section, the data is modeled by a two-site model (**TS**), we explain this model in detail in the following section. They provide an equation for the response ($r$), meaning the number of occupied sites per receptor, as follows:

$r=\frac{K_{1}L+2K_{1}K_{2}L^{2}}{1+K_{1}+K_{1}K_{2}L^{2}}$

To convert the response ($r$) to the proportion of occupied sites ($\boldsymbol{\theta}$), and compare the equilibrium ratios, we need first to renormalize dividing by 2 ($r$ is between 0 and 2, while $\boldsymbol{\theta}$ between 0 and 1). Then we need also to redefine $K_{1}$ and $K_{2}$ to its inverse values (notice they have magnitudes of 1/[Concentration]). This conversion leads to Eq. (S8).

$\boldsymbol{\theta}_{\mathbf{TS}}=\frac{\frac{K_{2}}{2}L+L^{2}}{K_{1}K_{2}+K_{2}L+L^{2}}$ (S8)

Equalizing Eqs. (2) and (S8) leads to a unique solution, given by Eqs (S9) and (S10).

$K_{1}=\frac{K}{2}$ (S9) $K_{2}=\frac{2K}{\omega}$ (S10)

The last result shows that **TS** and **NC** are equivalent, but the ratios are defined differently. In **NC**, it is the ratio between unbinding and binding rates $K=l/k$ and $\frac{K}{\omega}=l/\omega k$, while the **TS** model defines a unique ratio for each site, it simplifies the possibility of binding to one or the other site into just one ratio. The affinity of this ‘effective site’, which represents both equal binding sites, is two times the affinity of each **NC** sites (remember $K$ is the inverse of the affinity), and it does not represent the ratio of binding rates. This equivalence also explains the relation between $\omega$ and the ‘cooperative factor’ ($c$), used in literature, defined as the ratio of ratios expressed in Eq. (S11).

$c=\frac{K_{1}}{K_{2}}=\frac{\frac{K}{2}}{\frac{2K}{\omega}}=\frac{\omega}{4}$ (S11)

To do the same now with **IB**, we equalize Eqs. (1) and (S8) and get again a unique solution, given by Eqs. (S12) and (S13). Notice that $K_{1}$ and $K_{2}$ represent, again, effective sites and they are not the ratios of the unbinding and binding rates.

$K_{1}=\frac{K_{10}K_{01}}{K_{10}{+K}_{01}}$ (S12) $K_{2}=K_{10}{+K}_{01}$ (S13)

It is also possible to compare the **TS** model with a binding model of a single site, equalizing Eqs. (S8) and (S14) (the Michaelis-Menten curve **MM**, for just one site) and get a unique solution again, given by Eqs. (S15) and (S16). Notice that $K_{1}$ and $K_{2}$ represent, again, effective sites and they are not the ratios of the unbinding and binding rates. However, there is a difference in this case, notice that Eqs. (S15) and (S16) imply Eq. (S17), meaning that **TS** model can be equivalent to a single site model with ratio $K$ but only if there is a specific relation between $K_{1}$ and $K_{2}$. This reduces the two degrees of freedom of the **TS** model into just one, consistently with the fact that we are comparing a model with two sites with another that has only one. The restriction imposed by Eq. (S17), and considering Eq. (S11), means both sites are equivalent.

$\boldsymbol{\theta}_{\mathbf{MM}}=\frac{L}{K+L}$ (S14)

$K_{1}=K/2$ (S15) $K_{2}=2K$ (S16) $4K_{1}=K_{2}$ (S17)

In summary, relations (S9), (S10), (S12), (S13), (S15) and (S16) allow to compare the different ratios for fittings with different models. Table 1 in the main text shows these comparisons for three of the four experimental datasets of Fig 7 in the main text, Equal Sites, **NC** and **IB**, in the case of different and cooperative sites, ratios are not provided. The two values shown for each $K$,$K/\omega$, $K_{10}$ and$K_{01}$ are, on one hand, from fitting the extracted original data from each paper with our respective model (**NC** or **IB**). On the other hand, from the reported values of $K_{1}$ and $K_{2}$ in each paper (or just $K$ in the Equal Sites example). In this cases, the inverse relations of Eqs. (S9) and (S10) or (S12) and (S13) were used to infer $K$ and $K/\omega$ or $K_{10}$ and$K_{01}$ respectively from the reported $K_{1}$ and $K_{2}$ or $K$ values. The expressions in Eqs. (S18-S21) are these inverse relations.

$K=2K_{1}$ (S18) $K/\omega=\frac{K_{2}}{2}$ (S19)

$K_{10}=\frac{K_{2}}{2}\left( 1+\sqrt{\left( 1-4\frac{K_{1}}{K_{2}} \right)} \right)$ (S20) $K_{01}=\frac{K_{2}}{2}\left( 1-\sqrt{\left( 1-4\frac{K_{1}}{K_{2}} \right)} \right)$ (S21)

## 2.3 The Two-Site Model (TS).

### 2.3a Comparison with IB and NC

**TS** is a widely used model for a receptor with two binding sites, as **NC** and **IB**. In Fig H we compare the three models binding schemes. The principal difference between **TS** and **IB** and **NC** is that, in **TS**, the double occupation of the receptor occurs always going through the same and unique pathway where there is a first binding with its own binding $\left( k_{1} \right)$ and unbinding $\left( l_{1} \right)$ rates and ratio $\left( K_{1}=l_{1}/k_{1} \right)$, and a second one, with $k_{2},l_{2}$ and $K_{2}$. Consequently, as the parameters allow setting different affinities to the first and second bindings, **TS** is analogous to **NC** and not to **IB**, where the first binding can occur to the less affine site and the second to the more affine site.

**
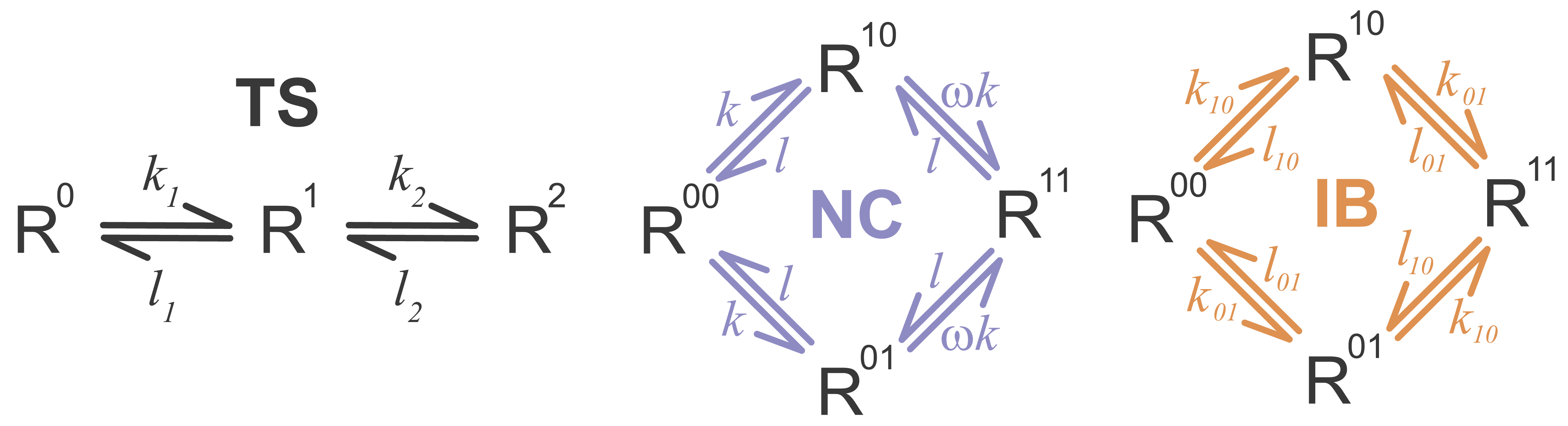
**

**Fig H:** The three models binding schemes and parameters. Left TS, middle NC and right IB.

### 2.3b Analogy between TS and NC

The analogy between **TS** and **NC** can be mathematically defined. Using the law of mass action, we obtain the sets of differential equations Eqs. (S22) and (S23) for **TS** and **NC** respectively.

$\left( \begin{matrix} \frac{dR^{0}}{dt}= -k_{1}LR^{0}+l_{1}R^{1} \\ \frac{dR^{1}}{dt}=k_{1}LR^{0}-l_{1}R^{1}-k_{2}LR^{1}+l_{2}R^{2} \\ \frac{dR^{2}}{dt}=k_{2}LR^{1}-l_{2}R^{2} \end{matrix} \right)$ (S22) $\left( \begin{matrix} \frac{dR^{00}}{dt}= lR^{10}-kLR^{00}+lR^{01}-kLR^{00} \\ \frac{dR^{10}}{dt}= kLR^{00}-lR^{10}-\omega kLR^{10}+lR^{11} \\ \begin{matrix} \frac{dR^{01}}{dt}= kLR^{00}-lR^{01}-\omega kLR^{01}+lR^{11} \\ \frac{dR^{11}}{dt}=\omega kLR^{10}+\omega kLR^{01}-2lR^{11} \end{matrix} \end{matrix} \right)$ (S23)

Notice that the main difference mentioned before is reflected in the different number of variables for each model. While **TS** has only 3 variables ($R^{0},R^{1}$and$R^{2}$), **NC** has 4 ($R^{00},R^{10},R^{01}$and$R^{11}$). This difference arise directly from making the distinction or not between the intermediate half occupied receptor states. However, we can reduce the 4 equations system of **NC** to a just 3 equations system by grouping $R^{10}$ and $R^{01}$ and taking the sum of its derivatives. This reduction leads from Eqs. (S23) to Eqs. (S24). A simple comparison of Eqs. (S24) and (S22) reveals that they are the same mathematical system of equations, with just different variables and parameters definitions. Nevertheless, this comparison allows also defining the relations between them, Eqs. (S25-S27) for variables and Eqs. (S28-S31) for parameters.

$\left( \begin{matrix} \frac{dR^{00}}{dt}= -2kLR^{00}+l\left( R^{10}+R^{01} \right) \\ \frac{d\left( R^{10}+R^{01} \right)}{dt}= 2kLR^{00}-l\left( R^{10}+R^{01} \right)-\omega kL\left( R^{10}+R^{01} \right)+2lR^{11} \\ \frac{dR^{11}}{dt}=\omega kL\left( R^{10}+R^{01} \right)-2lR^{11} \end{matrix} \right)$ (S24)

$R^{00}=R^{0}$ (S25) $R^{10}+R^{01}=R^{1}$ (S26) $R^{11}=R^{2}$ (S27)

$2k=k_{1}$ (S28) $\omega k=k_{2}$ (S29)

$l=l_{1}$ (S30) $2l=l_{2}$ (S31)

Notice that the analogy implies that the intermediate unique **TS** state $R^{1}$ has to be the sum of both intermediate states $R^{10}$ and $R^{01}$ (Eq. (S26)), while the two other states of the receptor, empty ($R^{0}$) or fully occupied ($R^{1}$), are just equal to $R^{00}$ and $R^{11}$ respectively (Eqs. (S25) and (S27)). This unique path of occupation proposed in the **TS** model has consequences also in the binding and unbinding rates. The first binding rate of **TS** needs to be equal to two times the one in **NC** (Eq. (S28)), and this is also coherent to the fact than the probability of binding to one or another site is the double of binding to just one of the two. The same happens with the second unbinding rate (Eq. (S31)), whereas the unbinding can occur in any of each site in **NC**, in **TS** it only occurs in the unique second site but with double probability. This difference in rates does not occur for the parameters related to the intermediate states bindings and unbindings (Eqs. (S29) and (S30)). In these cases, the rates of **NC** and **TS** are equal because, in **NC**, the binding or unbinding can happen in any of the two intermediate states, and as the sum of them equals the unique intermediate state of **TS**, the amount of bindings and unbindings remains the same without the need to double parameters.

### 2.3c Degrees of freedom

Despite the analogy between **TS** and **NC**, **TS** has 4 parameters ($k_{1},k_{2},l_{1}$ and $l_{2}$) while **NC** has 3 ($k,l,\omega$). Then, **TS** has one degree of freedom more than **NC**. Notice than this arises from Eqs. (S30) and (S31), which imply that $2l_{1}=l_{2}$ for the analogy. In conclusion, **TS** is more general than **NC**, and we can equally reproduce any dynamics obtained by **NC** with **TS**, just choosing the parameters according to Eqs. (S28-S31). However, we can obtain dynamics from **TS** that cannot be reproduced by **NC**. These are just those arising from any set of parameters where $2l_{1}\neq l_{2}$.

### 2.3d Analogy between TS and IB is not possible

A natural question than arises is if a similar analogy between **TS** and **IB** can be made. The answer is no, because **TS** allows to have different affinities between the first and second bindings, but not between one and the other site. **TS** cannot represent an example where a first binding occurs to the less affine site. Mathematically, if we want to do the same as before with **NC**, we can write the two systems of differential equations (Eqs. (S32) and (S33)), sum and try to group the intermediate states of **IB** (Eq. (S32)). However, is not possible to replace $R^{10}+R^{01}$ and $R^{1}$ to obtain the same systems of equations, because each variable $R^{10}$ and $R^{01}$ is multiplied by a different parameter in each term. In conclusion, the analogy cannot be done.

$\left( \begin{matrix} \frac{dR^{0}}{dt}= -k_{1}LR^{0}+l_{1}R^{1} \\ \frac{dR^{1}}{dt}=k_{1}LR^{0}-l_{1}R^{1}-k_{2}LR^{1}+l_{2}R^{2} \\ \frac{dR^{2}}{dt}=k_{2}LR^{1}-l_{2}R^{2} \end{matrix} \right)$(S32) $\left( \begin{matrix} \frac{dR^{00}}{dt}= l_{10}R^{10}-k_{10}LR^{00}+l_{01}R^{01}-k_{01}LR^{00} \\ \frac{dR^{10}}{dt}= k_{10}LR^{00}-l_{10}R^{10}-k_{01}LR^{10}+l_{01}R^{11} \\ \begin{matrix} \frac{dR^{01}}{dt}= k_{01}LR^{00}-l_{01}R^{01}-k_{10}LR^{01}+l_{10}R^{11} \\ \frac{dR^{11}}{dt}=k_{01}LR^{10}+k_{10}LR^{01}-l_{01}R^{11}-l_{10}R^{11} \end{matrix} \end{matrix} \right)$ (S33)

$\left( \begin{matrix} \frac{dR^{00}}{dt}= -\left( k_{10}+k_{01} \right)LR^{00}+l_{01}R^{01}+ l_{10}R^{10} \\ \frac{d\left( R^{10}+R^{01} \right)}{dt}=\left( k_{10}+k_{01} \right)LR^{00}-\left( l_{10}+k_{01}L \right)R^{10}-\left( l_{01}+k_{10}L \right)R^{01}+\left( l_{10}+l_{01} \right)R^{11} \\ \frac{dR^{11}}{dt}=k_{01}LR^{10}+k_{10}LR^{01}-\left( l_{10}+l_{01} \right)R^{11} \end{matrix} \right)$ (S34)

### 2.3e Equilibrium dose-response curves

We can deduce the equilibrium dose-response curve of **TS** from Eq (S32) and the conservation of total receptor (Eq (S35)). Just equalizing the first and last derivatives to zero, we get the expressions of Eqs. (S36) and (S37) respectively.

$R^{0}+R^{1}+R^{2}=R_{0}$ (S35) $R^{0}=\frac{K_{1}}{L}R^{1}$ (S36) $R^{2}=\frac{L}{K_{2}}R^{1}$ (S37)

The dose response curve, defined in the same terms as for **IB** and **NC** (Eqs. (1) and (2)), is the proportion of occupied sites versus the ligand concentration. The proportion of occupied sites ($\boldsymbol{\theta}_{\mathbf{TS}}$) is one for each intermediate state and two for the double occupied state divided the total amount of sites, which is two times the amount of receptors (Eq. (S38)). Replacing Eqs. (S35-S37) in Eq. (S38) leads to Eq. (S39). Notice this is the same equilibrium dose-response curve of Eq. (S8) derived from the literature. It is also the same we used to compare the all models and obtain the relations between the equilibrium constants (Eqs. (S18-S21)).

$\boldsymbol{\theta}_{\mathbf{TS}}=\frac{R^{1}+2R^{2}}{2R_{0}}$ (S38) $\boldsymbol{\theta}_{\mathbf{TS}}=\frac{\frac{K_{2}L}{2}+L^{2}}{K_{1}K_{2}+K_{2}L+L^{2}}$ (S39)

### 2.3f Conclusions

The conclusion of the **TS** model analysis is that is similar and even more general than **NC**, and it is correct to use it to analyze dynamics of a two equal sites receptor with positive or negative cooperativity, but keeping in mind that $K_{1}$ and $K_{2}$ do not represent ratios of unbinding and binding rates. However, if both binding sites are not equal, it is mathematically correct to fit an equilibrium dose-response curve, but because of the indistinguishability. The use of **TS** to model the dynamics of a receptor with two different sites is incorrect.

# 3. All the parameter values used in the paper and this supplement.

All the parameter values used in this paper to obtain different curves of Fig 4, 5, 6A in the main text and Fig B are showed in Table A.

| **Set** | **Parameters** | | | | | | | **Equilibrium Ratios** | | | |
| --- | --- | --- | --- | --- | --- | --- | --- | --- | --- | --- | --- |
|  | *k_10_* | *k_01_* | *l_10_* | *l_01_* | *k* | *l* | ω | K_10_ | K_01_ | K | K/ω |
|  | **Fig 4 in main text datasets and fitted parameters** | | | | | | | | | | |
| **Dataset** | **0.026** | **0.022** | **4.1** | **8.0** |  |  |  | **0.85** | **1.9** |  |  |
| **IB fit** | **4.1** | **8.0** | **0.026** | **0.022** |  |  |  | **2.0** | **0.82** |  |  |
| **NC fit** |  |  |  |  | **0.20** | **0.24** | **0.84** |  |  | **1.2** | **1.4** |
|  | **Fig 5 in main text example datasets** | | | | | | | | | | |
| **1** |  |  |  |  | **0.40** | **0.11** | **0.38** |  |  | **0.28** | **7.2** |
| **2** | **0.026** | **0.022** | **4.1** | **8.0** |  |  |  | **0.85** |  |  |  |
| **3** | **53** | **6.8** | **10** | **0.098** |  |  |  | **0.13** |  |  |  |
| **4** |  |  |  |  | **0.016** | **0.40** | **0.17** |  |  | **25** | **150** |
| **5** |  |  |  |  | **5.0** | **8.7** | **0.45** |  |  | **1.7** | **3.9** |
| **6** | **0.22** | **0.012** | **2.6** | **14** |  |  |  | **0.055** | **5.4** |  |  |
| **7** | **0.12** | **0.028** | **0.073** | **0.044** |  |  |  | **0.23** | **0.6** |  |  |
|  | **Fig 6A in main text example datasets** | | | | | | | | | | |
| **Dataset** |  |  |  |  | **5.0** | **8.7** | **0.45** |  |  | **1.7** | **3.9** |
|  | **Fig B regimes** | | | | | | | | | | |
| **Biphasic** | **10^-1^** | **10^-3^** | **10^2^** | **10^6^** |  |  |  | **10^-2^** | **10^4^** |  |  |
| **Decreasing** | **10^7^** | **10^5^** | **10^-4^** | **10^0^** |  |  |  | **10^-2^** | **10^4^** |  |  |
| **Increasing** | **10^2^** | **10^0^** | **10^2^** | **10^6^** |  |  |  | **10^-2^** | **10^4^** |  |  |

**Table A:** Parameter values and equilibrium ratios for each set shown in the paper. **Top rows**: deterministic numerical simulations in Fig 4 in the main text (it is an **IB** example) and both fits with **IB** and **NC**, notice the fit is perfect with **IB**, the only difference is that sites are swapped, but **IB** is symmetric on swapping binding sites. **Middle rows**: stochastic numerical simulations in Fig 5 and in Fig 6A in the main text. **Bottom rows**: **IB** examples in Fig B to show the three different $\mathbf{DynR}\left( t \right)$ regimes. Notice that parameters were chosen to maintain the same equilibrium ratios.

# 4. References

1. Maddala GS. Introduction to Econometrics. 2nd ed. Macmillan Publishing Company; 1992. pp. 165–170

2. Zhao C, Ren J, Gregoliński J, Lisowski J, Qu X. Contrasting enantioselective DNA preference: Chiral helical macrocyclic lanthanide complex binding to DNA. Nucleic Acids Res. 2012;40(16):8186–96.

3. Myszka DG, Arulanantham PR, Sana T, Wu Z, Morton TA, Ciardelli TL. Kinetic analysis of ligand binding to interleukin-2 receptor complexes created on an optical biosensor surface. Protein Sci. 1996;5(12):2468–78.

4. Watson LC, Kuchenbecker KM, Schiller BJ, Gross JD, Pufall MA, Yamamoto KR. The glucocorticoid receptor dimer interface allosterically transmits sequence-specific DNA signals. Nat Struct Mol Biol. 2013;20(7):876–83.
